# Reproductive Disorders in Pet Reptiles Presented to a Specialized Exotic Animal Referral Center in Northern Italy (2020–2023)

**DOI:** 10.3390/ani16182930

**Published:** 2026-09-18

**Authors:** Alessandro Vetere, Martina Gavezzoli, Michela Ablondi, Giordano Nardini, Francesco Di Ianni

**Affiliations:** 1 Department of Veterinary Medical Sciences, University of Parma, 43126 Parma, Italy; 2 Exotic Service, Clinica Veterinaria Modena Sud, Spilamberto, 41057 Modena, Italy

**Keywords:** reptiles, reproduction, pathology, squamata, chelonia, follicular stasis

## Abstract

Reproductive disorders display an important, often understudied aspect of veterinary clinical practice in reptiles. The present study provides an overview of such conditions analyzing their prevalence, clinical manifestations, and therapeutic management in reptiles (Testudines and Squamata) presented by private owners to a specialized exotic animal clinic in northern Italy. A retrospective analysis of 646 reptile cases reported between 2020 and 2023 revealed that reproductive disorders affecting both male and female reptiles accounted for 11.11% of all clinical cases. The most frequently diagnosed conditions were phallus prolapse (31.91%, 15/47), pre-ovulatory follicular stasis (POFS; 19.15%, 9/47), and post-ovulatory egg stasis (POES; 14.89%, 7/47), all predominantly managed through surgical intervention. The often-non-specific nature of clinical signs makes early diagnosis challenging and may delay treatment. Overall, 85.11% (40/47) of affected reptiles had a positive clinical outcome. These findings provide clinically relevant information on the prevalence, presentation, and management of reproductive disorders in captive reptiles and may support earlier diagnosis and improved therapeutic decision-making in clinical practice.

## Simple Summary

Reproductive disorders are common but often overlooked health problems in captive reptiles. This study reviewed 646 reptile cases presented to a specialized veterinary clinic in northern Italy between 2020 and 2023. Reproductive disorders were diagnosed in 11.11% of the cases, with phallus prolapse, pre-ovulatory follicular stasis, and post-ovulatory egg stasis being the most frequent conditions. Most affected reptiles were treated surgically, and 85.11% had a positive outcome. These findings highlight the importance of early recognition and appropriate treatment of reproductive disorders to improve the health and welfare of captive reptiles.

## 1. Introduction

The popularity of reptiles as companion animals has increased markedly over the last two decades. Surveys from the American Veterinary Medical Association [1] and European pet ownership studies [2] indicate that millions of households now keep reptiles, with ownership trends rising faster than for traditional small mammals and birds. The growing number of captive reptiles has resulted in a parallel increase in clinical presentations of husbandry-related diseases, particularly disorders of the reproductive system [3,4]. Reproductive pathologies are considered a major and often underdiagnosed issue in reptile medicine [3,4,5,6,7,8,9]. These conditions encompass a broad range of entities, including pre-ovulatory follicular stasis, post-ovulatory egg stasis, dystocia, oviductal obstruction, infertility, cloacal and hemipenal disorders, ovarian torsion, and yolk coelomitis [9,10,11,12]. Many reproductive disorders in reptiles have been associated with inadequate husbandry, including inappropriate environmental management, nutritional imbalances, and systemic diseases, all of which represent important predisposing factors in captive reptiles [12,13,14,15,16,17,18,19,20,21,22]. Although individual reproductive disorders have been extensively described in the veterinary literature, most publications consist of single case reports or narrative reviews focused on specific conditions or species [3,10,11]. Comprehensive retrospective studies evaluating the prevalence, distribution, treatment, and clinical outcome of reproductive disorders across different reptile species remain limited. Consequently, there is still a lack of clinical data describing the occurrence of these disorders in pet reptiles presented to referral veterinary hospitals. The aim of this retrospective study was to describe the prevalence and distribution of reproductive disorders in pet reptiles (Testudines and Squamata) presented to a specialized exotic animal referral center in northern Italy (Exotic Animal Service, Clinica Veterinaria Modena Sud, Spilamberto, Emilia-Romagna) between January 2020 and January 2023. In addition, the study summarizes the most common reproductive disorders, their clinical presentation, treatment, and clinical outcome, providing an overview of their occurrence in a referral clinical setting.

## 2. Materials and Methods

In the present study, data were collected between 2020 and 2023. The study included medical records of pet reptiles (Testudines and Squamata) presented to the Exotic Animal Service, Clinica Veterinaria Modena Sud, Spilamberto, Italy, between 2020 and 2023. Records meeting the inclusion criteria were filtered and organized into a Microsoft Excel file (Microsoft Corporation, Redmond, WA, USA), divided into four-month periods, and subsequently unified to create a clear timeline of clinical cases. For each patient, the following information was collected: date of admission, species, sex (when known), age (when available), clinical history, clinical examination findings, diagnostic tests performed, final diagnosis, treatment, and clinical outcome. Those clinical examinations, when indicated, included hematology, serum biochemistry, fecal examination, cytology, toxicology, histopathology, diagnostic imaging (radiography, ultrasonography, computed tomography), endoscopy, microbiological examinations, and post-mortem examination in animals that died and for which owner consent for necropsy was obtained. Based on the clinical and diagnostic findings, reproductive disorders were classified as hemipenile/phallus prolapse in males, and pre-ovulatory follicular stasis (POFS), post-ovulatory egg stasis (POES), dystocia, yolk coelomitis, oviduct prolapse, ectopic eggs, or ovarian torsion in females. Treatments were classified into four categories: medical treatment, surgical treatment, euthanasia, and no treatment. Medical treatment included pharmacological and supportive therapies, whereas surgical treatment comprised minimally invasive and open surgical procedures performed under general anesthesia. Euthanasia was performed with the owner’s consent when considered clinically appropriate. Clinical outcome was classified as positive when treatment resulted in disease resolution or long-term stabilization with an acceptable quality of life, negative when the patient died despite treatment, and unknown when follow-up information was unavailable at the end of the study.

### Data Analysis

The statistical analysis and graphical visualization of the data were conducted using R (version 4.4.1; R Foundation for Statistical Computing, Vienna, Austria). The packages tidyverse, dplyr, and readxl were used for data processing, while ggplot2 was employed for graphical representation. The association between species and diseases was further investigated using standardized residuals, calculated using the vcd and nnet packages. Several graphs were produced to represent disease prevalence across species and to identify potential trends or distribution patterns. The association between species and diseases was assessed using a chi-square test of independence. Statistical significance was set at *p* < 0.05. Standardized residuals were subsequently examined to identify cells contributing substantially to the observed association, with absolute residual values greater than 2 considered indicative of a significant deviation from the expected frequency. For the analysis of disease distribution among species, only species represented by at least two individuals among the clinical cases were included.

## 3. Results

During the period between 2020 and 2023, a total of 646 reptiles were admitted to the exotic animal service of a clinic in northern Italy. From those, 223 (34.52%) were healthy animals undergoing routine procedures and were therefore excluded from further analysis. The remaining 65.48% (423/646) corresponded to affected reptiles admitted to the clinic. Among the others 423 (65.48%), corresponded to affected reptiles admitted to the clinic, being 216 (51.06%) from Testudines Order, and 207 (48.94%) to Squamata order.

**Figure 1:**
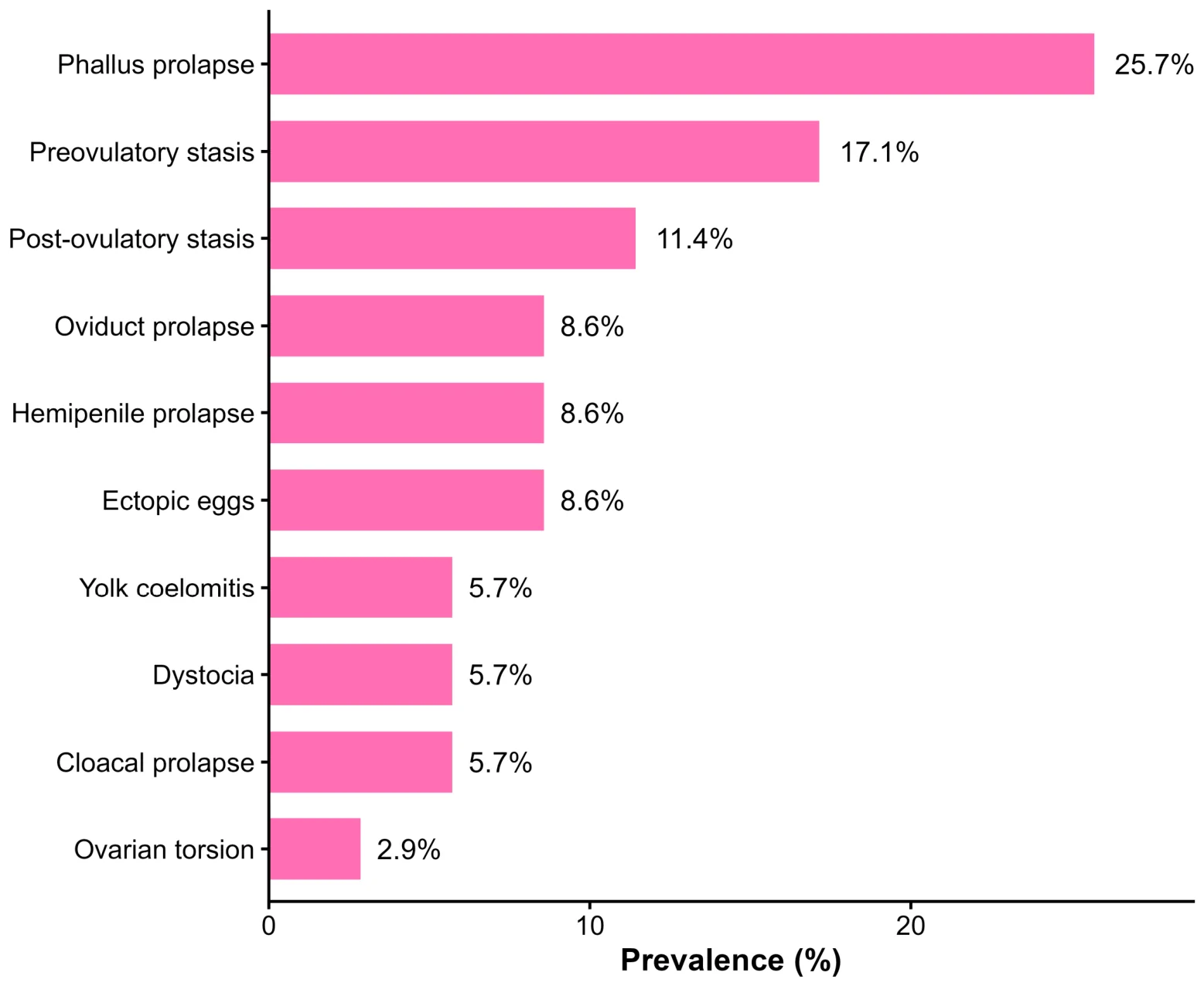
Overall prevalence of type of diagnosis in reproductive diseases by the Exotic Animal Service at the Clinica Veterinaria Modena Sud in Spilamberto, Italy.

From the retrospective study of the data, it was found that 47 animals were admitted to the clinic with pathologies related to the reproductive system. The reported diagnoses show that 31.91% (15/47) of cases presented with prolapse of phallus-hemipenes, 19.15% (9/47) with pre-ovulatory stasis, 14.89% (7/47) with post-ovulatory stasis, 8.51% (4/47) with dystocia, 8.51% (4/47) with yolk coelomitis, 8.51% (4/47) with oviduct prolapse, 6.38%,(3/47) with ectopic eggs, and 2.13% (1/47) with ovarian torsion (Figure 1).

Regarding pathologies of reproductive system, it was found that 47 animals were admitted with different conditions, being 15 (31.91%) with prolapse of phallus-hemipenes, 9 (19.15%) with pre-ovulatory stasis, 7 (14.89%) with post-ovulatory stasis, 4 (8.51%) with dystocia, 4 (8.51%) with yolk coelomitis, 4 (8.51%) with oviduct prolapse, 3 (6.38%) with ectopic eggs and 1 (2.13%) with ovarian torsion.

**Figure 2:**
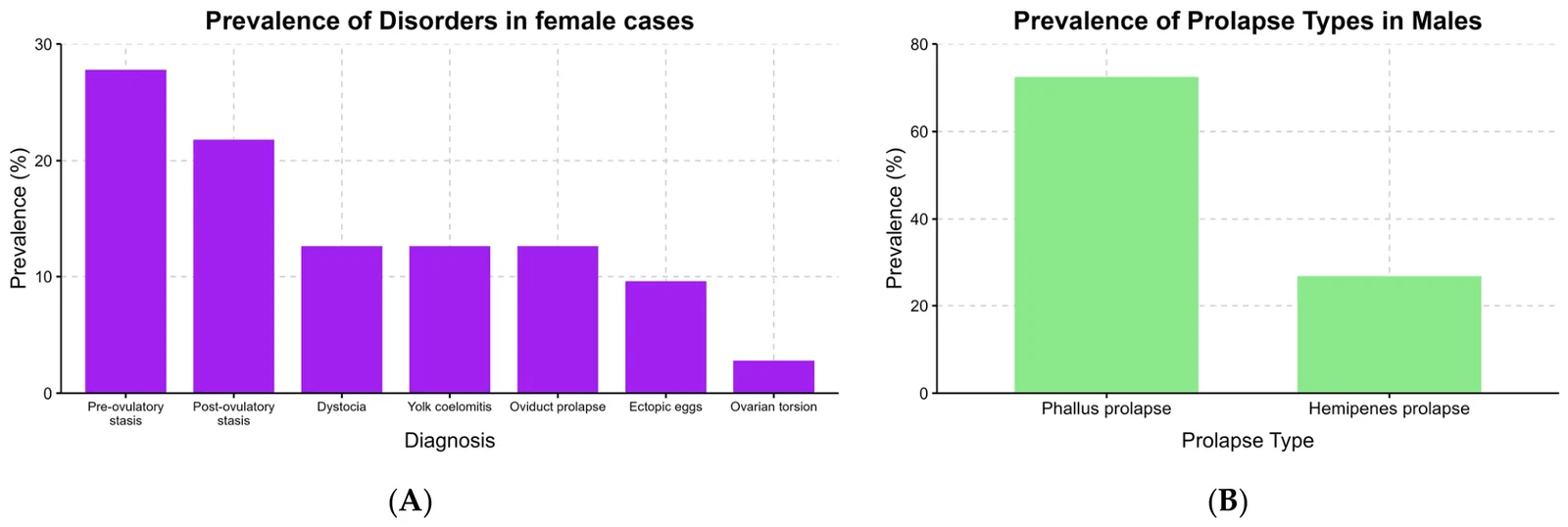
(**A**,**B**) Prevalence of type of diagnosis divided by sex.

Males present as their only condition the prolapse of the copulatory organ, distributed as follows between the orders Testudines and Squamata: 11/15 (73.33%) phallus prolapse in Testudines, 4/15 (26.67%) hemipenes prolapse in Squamata (Figure 2B).

The distribution of accesses in different months of animals displaying this disorder was also analyzed with the following results: no accesses were reported in January, February, June and November. 2/15 (13.33%) of animals were admitted to the clinic because of this disorder in March, 1/15 (6.67%) in April, 3/15 (20%) in May, 3/15 (20%) in July, 4/15 (26.67%) in August, 1/15 (6.67%) in September, and finally 1/15 (6.67%) 1/15 in December.

**Figure 3:**
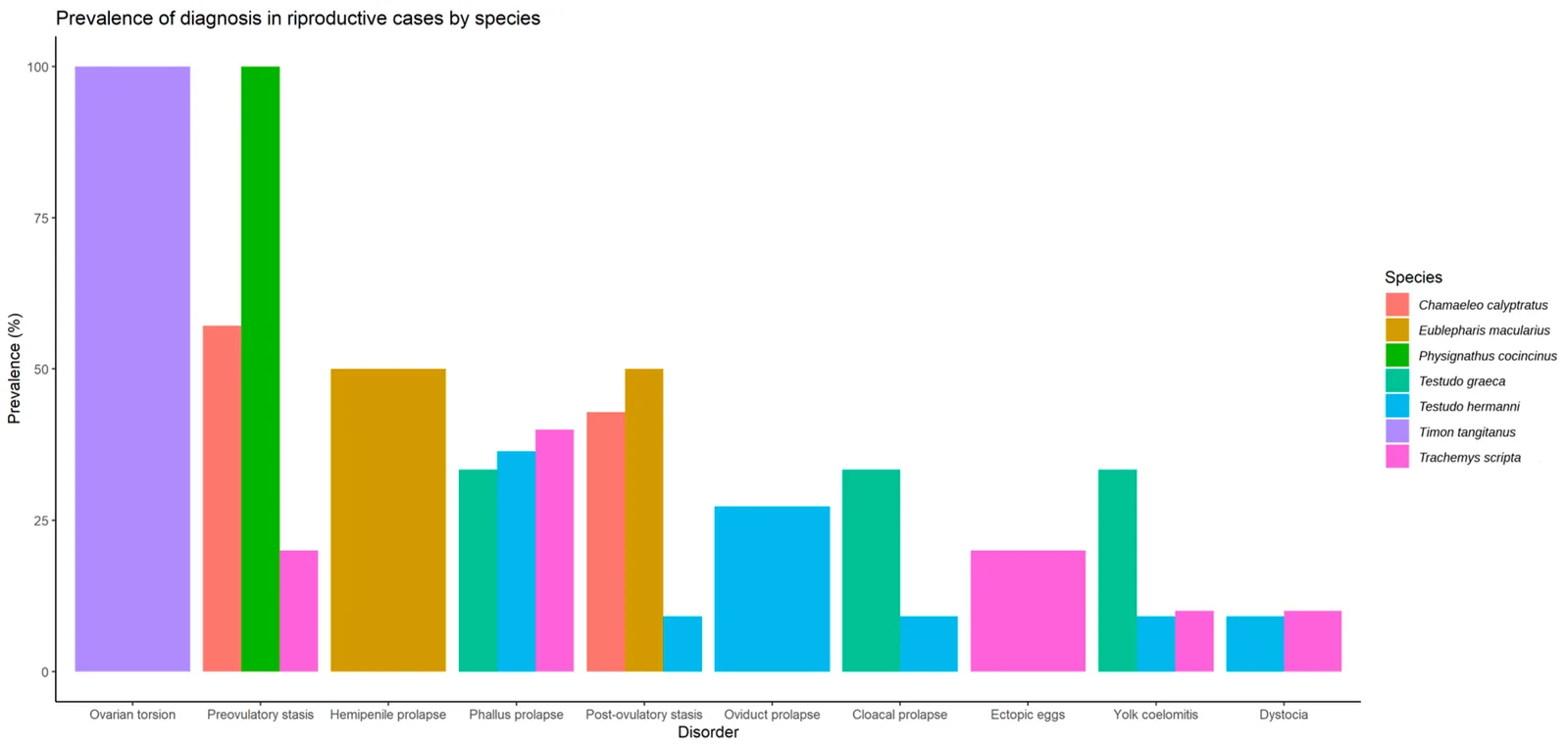
Prevalence of type of diagnosis divided by seven species.

Only species with a minimum of two individuals among the clinical cases were considered, as this provides greater statistical significance Figure 3. *Physignathus cocincinus* predominantly displayed pre-ovulatory stasis, while *Chamaeleo calyptratus* showed a predominance of pre-ovulatory stasis, followed by post-ovulatory stasis. In *Eublepharis macularius*, hemipenile prolapse and post-ovulatory stasis were observed with similar frequency. *Testudo hermanni* displayed a more heterogeneous distribution of diagnoses, with phallus prolapse and oviduct prolapse being the most frequently observed conditions, followed by post-ovulatory stasis, dystocia, and yolk coelomitis. Similarly, *Trachemys scripta* displayed phallus prolapse as the most frequently observed diagnosis, followed by pre-ovulatory stasis and ectopic eggs, while dystocia and yolk coelomitis were less frequently observed. *Testudo graeca* displayed phallus prolapse more frequently than yolk coelomitis.

**Figure 4:**
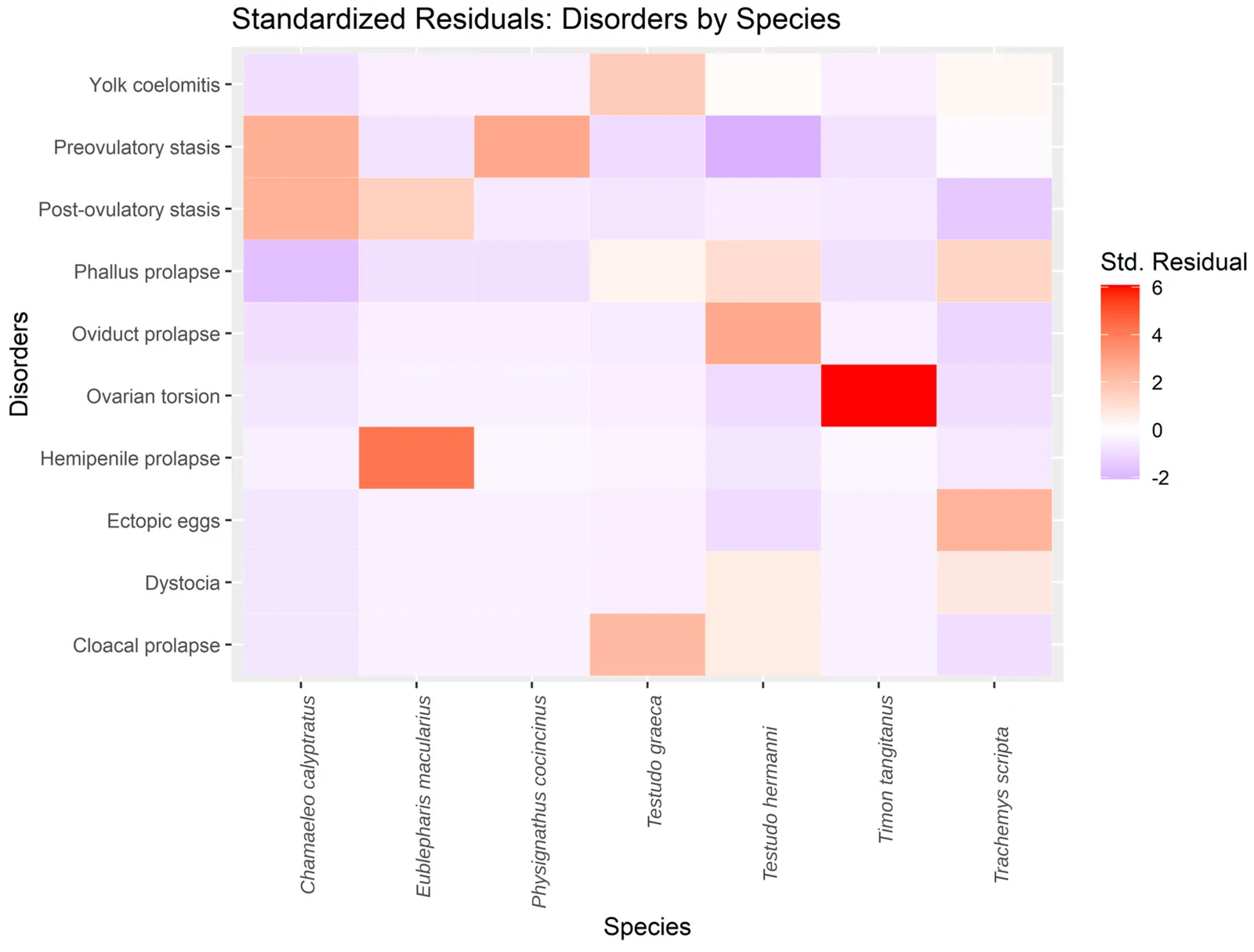
Standardized residual of the disorder divided by species.

A statistically significant association was observed between species and the incidence of specific diseases (Figure 4).The Pearson’s chi-square test revealed a statistically significant association between species and disease distribution (χ^2^ = 103.32, df = 54, *p* = 6.159 × 10^−5^). Examination of the standardized residuals identified several species–disease combinations that deviated substantially from the expected frequencies (Figure 4). Positive residuals greater than 2 indicated higher-than-expected frequencies, whereas negative residuals below −2 indicated lower-than-expected frequencies. The strongest positive deviation was observed for ovarian torsion in the Moroccan eyed lizard (*Timon tangitanus*; standardized residual = 6.08), followed by hemipenile prolapse in the leopard gecko (*E. macularius*; 4.24). Other positive deviations were observed for oviduct prolapse in the Hermann’s tortoise (*T. hermanni*; 2.78), pre-ovulatory stasis in the Chinese water dragon (*P. cocincinus*; 2.77), pre-ovulatory stasis (2.54) and post-ovulatory stasis (2.52) in the veiled chameleon (*C. calyptratus*), and ectopic eggs in the red-eared slider (*T. scripta*; 2.39). In contrast, pre-ovulatory stasis in *T. hermanni* showed a negative residual (−2.08), indicating a lower-than-expected frequency. These deviations suggest that specific species–disease combinations contributed substantially to the significant association observed between species and disease distribution.

### 3.1. Clinical Data

For each individual, the following information was considered: species (scientific name), order, sex, age, results of the clinical examination, diagnosis, type of treatment and the outcome.

### 3.2. Treatment

Of the 47 individuals analyzed for reproductive pathologies, 45 received a treatment, one was euthanized, and the remaining one did not receive any treatment. Of the patients who received treatment, 38 underwent surgical procedures, while 7 were treated medically.

### 3.3. Outcome

Overall, it was observed that among the clinical cases analyzed, 40/47 (85.11%) survived, while 7/47 (14.89%) died. In detail, 5/47 (62.50%) of cases had a negative outcome despite receiving treatment, when 2/47 (37.50%) were not treated, as they were either found dead by the owner or euthanized.

## 4. Discussion

In this retrospective study, reproductive disorders were more frequently diagnosed in Testudines than in Squamata (13.43% vs. 9.18% of clinical cases), although they represented an important cause of presentation for both orders. This finding may suggest a predisposition of this group to reproductive tract diseases, and indeed the literature clearly shows that in this Order such pathologies are far from uncommon [8].

### 4.1. Phallus/Hemipenes Prolapse

Phallus or hemipenile prolapse is characterized by persistent protrusion of the copulatory organ from the cloaca and is primarily reported in sexually mature males [23], consistent with the present study, in which all individuals of known age were at least three years old. The condition was most frequently observed in chelonians, particularly *T. hermanni*, *T. scripta*, *T. graeca*, and *Testudo marginata*, whereas snake species accounted for most squamate cases. Previous studies have identified trauma, mating-related injuries, infections, gastrointestinal or cloacal obstruction, metabolic disorders, and neurological disease as the main predisposing factors [22,24,25,26]. In the present case series, trauma, infectious processes, metabolic disorders, and cloacal obstruction were identified as the most common associated conditions, whereas no cases were associated with neurological disease. Clinical signs were generally nonspecific, with prolapse readily evident on physical examination; chronic cases frequently showed tissue necrosis, ulceration, or myiasis. Because most animals were presented with chronic or severely compromised prolapses, surgical amputation under general anesthesia was elected in all cases. This approach resulted in a positive outcome in all treated patients, supporting previous reports that surgery represents the treatment of choice in non-breeding reptiles when tissue viability is compromised [25,26,27,28,29].

### 4.2. Preovulatory Follicular Stasis (POFS)

Preovulatory follicular stasis (POFS) is characterized by retention of mature follicles within the ovary that fail to ovulate or regress. POFS is particularly common in captive reptiles [8,19,29], a finding supported by the present study, where it represented the most frequent reproductive disorder in females. The condition is described predominantly in lizards (especially iguanids, chameleons, monitor lizards, and agamids) and less frequently in chelonians, with few occurrences in snakes [26,30,31]. The species most frequently affected in this study were the veiled chameleon (*C. calyptratus*) and two Chinese water dragons (*P. cocincinus*), but chelonians were less frequently represented. The pathogenesis of POFS is multifactorial and includes environmental stressors, absence of a male, inadequate diet, endocrine dysfunction, systemic illness, ovarian cysts, oophoritis, or neoplasia [8,25]. In the present study, most cases were associated with sepsis, while one case was linked to nutritional secondary hyperparathyroidism (NSHP). Clinical signs included anorexia, lethargy, abdominal distension, and dehydration [26,32,33]. However, these signs are nonspecific and may overlap with normal follicular development, particularly in early stages [25]. All treated animals underwent ovariosalpingectomy and survived, achieving full recovery. One animal was euthanized at the owner’s request. Surgical intervention is considered the treatment of choice and is typically followed by analgesics, anti-inflammatory medication, and antibiotics [26,29]. Prognosis is generally favorable [25]. Ovariectomy has also been proposed as a preventive measure. The use of exogenous hormones to regulate ovarian activity has been explored, although its efficacy across species remains unclear [26,31,34].

### 4.3. Post-Ovulatory Egg Stasis (POES)

POES, together with POFS, is one of the most common reproductive disorders in female reptiles, particularly lizards. POES represents a non-obstructive form of dystocia, characterized by the retention of normally ovulated eggs within the oviducts in the absence of a mechanical obstruction. It occurs predominantly in oviparous species, mainly regarding squamates, with a high prevalence reported in the veiled chameleon (*C. calyptratus*) [29,31]. POES is commonly associated with inadequate husbandry, including inappropriate nesting opportunities, suboptimal environmental conditions, nutritional imbalances, and hypocalcemia, all of which may impair normal oviposition [8,19,25,26,27,28,29]. As these factors were not systematically evaluated in this retrospective study, their specific contribution could not be assessed. Nevertheless, the favorable response to calcium supplementation and oxytocin observed in more than half of the cases suggests that functional rather than mechanical dystocia was present in a substantial proportion of affected animals. Conversely, patients requiring surgery were likely affected by more advanced or obstructive forms of egg retention, consistent with previous reports.

In the present study, 57.14% of cases were managed medically with oral fluid therapy, intramuscular calcium gluconate (100 mg/kg), and oxytocin (5 IU) [3,8]. The remaining 42.86% required surgical intervention, either via ovariosalpingectomy or less invasive endoscopy-assisted egg removal under general anesthesia. These findings are broadly in agreement with previous reports indicating that oxytocin tends to be more effective in chelonians than in squamates, where manual or endoscopy-assisted extraction is often required [25,26,28,29]. In contrast to this general trend, oxytocin was also administered to veiled chameleons (*C. calyptratus*) in the present series, suggesting that its clinical use in squamates may be more widespread than previously reported.

The prognosis for POES is generally favorable [25], a finding supported by this study, in which 85.71% of treated animals survived and recovered. One case, treated exclusively with medical management, resulted in therapeutic failure and death. This outcome raises questions about the limitations of conservative treatment in severe cases and highlights the potential need for timely surgical intervention in selected patients.

### 4.4. Yolk Coelomitis

Yolk coelomitis is characterized by the presence of yolk material within the coelomic cavity, leading to inflammation [8,26,32]. This condition has been described across reptilian taxa [32]. In the present study, all affected individuals belonged to the order Chelonia, specifically spur-thighed tortoise (*T. graeca*), red-eared slider (*T. scripta*), Hermann’s tortoise (*T. hermanni*), and stinkpot turtle (*Sternotherus odoratus*).

Yolk coelomitis is frequently secondary to other reproductive disorders, including follicular stasis, oophoritis, salpingitis, or dystocia. Clinical signs are often nonspecific and may include anorexia, lethargy, diarrhea, or reduced fecal and urine output. Iatrogenic causes have also been reported [8,19,26,32]. In our cases, the predominant signs were anorexia and lethargy; no cases of diarrhea or absence of feces or urine were documented.Treatment consisted of supportive care, ovariosalpingectomy, and coelomic lavage with sterile solution, combined with postoperative administration of analgesics, anti-inflammatory agents, and antibiotics. Although some authors report that ovaries and oviducts can occasionally be left in place [25], this practice is generally discouraged due to the risk of recurrence.

Despite appropriate management, the prognosis for yolk coelomitis is often guarded to poor, as yolk material in the coelom provides an excellent substrate for bacterial proliferation and sepsis [8,19,25]. Consistent with this, all cases in the present study resulted in death. In 50% of these, surgery could not be performed because the animals died before intervention.

Necropsy was performed in one non-surviving, non-operated case. Post-mortem examination confirmed the diagnosis, revealing abundant coelomic exudate consistent with yolk material and extensive fibrin deposition, suggesting a chronic disease process.

### 4.5. Oviduct Prolapse

Oviductal prolapse is characterized by the protrusion of a portion of the oviduct through the cloaca and is predominantly reported in snakes and chelonians [8,26]. In the present study, all cases involved chelonians, specifically Hermann’s tortoise (*T. hermanni*) This finding is likely influenced by the high prevalence of this species in captive collections in Italy, rather than reflecting a true species-specific predisposition. Although a definitive cause could not be identified in the cases examined, oviductal prolapse has been frequently reported in association with dystocia and other reproductive tract disorders, including salpingitis, follicular stasis, oviductal rupture, and neoplasia, with iatrogenic factors also potentially playing a role [25,26].

No clinical signs other than the visible prolapse were observed, which commonly indicates that the acute nature of the condition at presentation. In early or mild cases, clinical signs may be minimal, whereas more advanced cases can be associated with severe dehydration and sepsis [8]. Prolonged exposure of the tissue to air can result in desiccation and necrosis, requiring surgical excision [28]. In the present series, necrotic lesions were not observed, supporting the assumption that these were relatively recent prolapses [8].

All animals were treated surgically using two approaches widely described in the literature: organ repositioning and ovariosalpingectomy [8,26,28]. Corroborating with previous studies, non-severe cases were managed by careful cleansing of the prolapsed tissue, assessment for necrosis or ulceration, and repositioning. In the presence of edema, isotonic saline or dextrose solutions were applied prior to reduction, as previously suggest as suggested by Johnson (2019) [23] and Lubian et al. (2025) [3]. Ovariosalpingectomy was reserved for a single case with more extensive structural damage, resulting in permanent loss of reproductive function. All animals survived and achieved complete recovery.

### 4.6. Ectopic Eggs

Ectopic eggs are defined as eggs abnormally located within the coelomic cavity, colon, or urinary bladder and have been reported more frequently in chelonians [8]. In the present study, all cases involved chelonians, with ectopic eggs consistently founded within the urinary bladder. Ectopic egg formation is most often associated with dystocia but has also been reported in association with iatrogenic factors, including oxytocin administration [25,26]. According with these reports, all animals in the current study had a history of dystocia treated with oxytocin, further supporting the association between oxytocin therapy and ectopic egg deposition in the bladder.

Clinical signs associated with ectopic eggs are often absent or nonspecific and may include anorexia, dyspnea, nesting behavior without egg laying, hyperactivity, or lethargy [8]. In the present series, anorexia and lethargy were consistently observed, whereas other nonspecific signs were not recorded. Additionally, hindlimb paresis and cloacal discharge were noted and are more likely attributable to the underlying dystocia, as these signs are well recognized among its clinical manifestations [26].

Eggs located within the coelomic cavity generally require removal via celiotomy, whereas those in the urinary bladder can be removed by cystotomy or cystoscopy [26]. Cystoscopy is considered less invasive and has been associated with excellent outcomes 25. In line with these findings, cystoscopy was used in all cases in the present study for removal of bladder eggs, and the procedure was successful in every animal. Although ovariectomy or bilateral ovariosalpingectomy has been proposed in cases with severe oviductal compromise [25], these procedures were not required in any of the cases analyzed.

### 4.7. Ovarian Torsion

Ovarian torsion is considered a rare condition across animal species, including reptiles, with only a limited number of cases documented in the literature [11,15,32,33,34]. In Chelonia, a single case has been reported in the red-eared slider (*Trachemys scripta elegans*) [11]. In Squamata, sporadic cases have been described in the green iguana (*Iguana iguana*) [31,32], the panther chameleon (*Furcifer pardalis*) [33], and several gecko species (Gekkonidae) [34]. An additional case has been reported in the Moroccan eyed lizard (*T. tangitanus*), which was previously published by the authors as a case report and is included in the present retrospective analysis [10].

Previous reports suggest an association between ovarian torsion and other reproductive disorders, including egg retention, systemic bacterial infection [31], follicular stasis, and oophoritis [33]. Because of the severity and rapid progression of the condition, affected animals often die before treatment can be attempted [32]. When intervention is possible, surgical options include ovariectomy or bilateral ovariosalpingectomy [11,31].

Overall, the present study demonstrates that the prognosis of the most common reproductive disorders in reptiles is generally favorable when prompt diagnosis and appropriate treatment are achieved. Medical management was effective in selected cases, particularly in functional reproductive disorders, whereas surgical intervention remained the treatment of choice for advanced, obstructive, or irreversible conditions such as chronic phallus prolapse, severe dystocia, and ovarian disorders. Although several reproductive diseases are strongly influenced by husbandry, nutritional, and environmental factors, these variables were not systematically assessed in this retrospective study and warrant further investigation. Nevertheless, the findings provide clinically relevant information on the prevalence, presentation, treatment, and outcome of reproductive disorders in pet reptiles and may support earlier diagnosis and more informed therapeutic decision-making in clinical practice.

## 5. Conclusions

This research has contributed to a better knowledge of the occurrence of reproductive pathologies in reptiles within a clinical setting, highlighting both the therapeutic successes achieved in managing the most common conditions and the challenges that persist in the diagnosis and treatment of less well-characterized diseases. In addition, the study provides useful insights into the prevalence of specific reproductive disorders affecting many of the reptile species most commonly kept in captivity, as well as their associated clinical signs and outcomes. Further retrospective and prospective clinical studies including larger case series are needed to better characterize reproductive disorders in reptiles, refine diagnostic and therapeutic approaches, and support evidence-based clinical decision-making.

## Data Availability

The data presented in this study are available on request from the corresponding author.
